# Hydroxy(phenyl)pyruvic acid reductase in *Actaea racemosa* L.: a putative enzyme in cimicifugic and fukinolic acid biosynthesis

**DOI:** 10.1007/s00425-024-04382-6

**Published:** 2024-03-28

**Authors:** Anne Jahn, Maike Petersen

**Affiliations:** Institut für Pharmazeutische Biologie und Biotechnologie, Philipps-Universität Marburg, Robert-Koch-Str. 4, 35037 Marburg, Germany

**Keywords:** *Actaea racemosa* syn. *Cimicifuga racemose*, Cimicifugic acid, d-Isomer-specific 2-hydroxyacid dehydrogenase family (2HADH), Fukiic acid, Fukinolic acid, 4-Hydroxyphenyllactic acid, 4-Hydroxyphenylpyruvic acid, Photorespiration, Piscidic acid

## Abstract

**Main conclusion:**

Hydroxy(phenyl)pyruvic acid reductase from *Actaea racemosa* catalyzes dual reactions in reducing 4-hydroxyphenylpyruvic acid as well as β-hydroxypyruvic acid. It thus qualifies to be part of fukinolic and cimicifugic acid biosynthesis and also photorespiration.

**Abstract:**

The accumulation of fukinolic acid and cimicifugic acids is mainly restricted to *Actaea racemosa* (Ranunculaceae) and other species of the genus *Actaea*/*Cimicifuga*. Cimicifugic and fukinolic acids are composed of a hydroxycinnamic acid part esterified with a benzyltartaric acid moiety. The biosynthesis of the latter is unclear. We isolated cDNA encoding a hydroxy(phenyl)pyruvic acid reductase (GenBank OR393286) from suspension-cultured material of *A. racemosa* (ArH(P)PR) and expressed it in *E. coli* for protein production. The heterologously synthesized enzyme had a mass of 36.51 kDa and catalyzed the NAD(P)H-dependent reduction of 4-hydroxyphenylpyruvic acid to 4-hydroxyphenyllactic acid or β-hydroxypyruvic acid to glyceric acid, respectively. The optimal temperature was at 38 °C and the pH optimum at pH 7.5. NADPH is the preferred cosubstrate (K_m_ 23 ± 4 µM). Several substrates are accepted by ArH(P)PR with β-hydroxypyruvic acid (K_m_ 0.26 ± 0.12 mM) followed by 4-hydroxyphenylpyruvic acid (K_m_ 1.13 ± 0.12 mM) as the best ones. Thus, ArH(P)PR has properties of β-hydroxypyruvic acid reductase (involved in photorespiration) as well as hydroxyphenylpyruvic acid reductase (possibly involved in benzyltartaric acid formation).

## Introduction

*Actaea racemosa* L. (syn. *Cimicifuga racemosa*, black cohosh, Ranunculaceae) is native to North America. Extracts of rhizomes and roots of this herb have been used traditionally in folk medicine (Guo et al. 2017). In Europe, extracts from rhizomes of *A. racemosa* are well established for the relief of menopausal and menstrual irregularities (EMA/HMPC/48745/2017). Extracts from plant organs of different *Actaea* species are used for the treatment of various diseases, e.g., head- and toothache, sore throat, uterine prolapse. Whereas extracts of the genus *Actaea* are used for the treatment of a broad variety of diseases, the use of *A. racemosa* (extracts from rhizomes and roots) is restricted to treat hot flushes, nervousness, depressive moods, and sleep disorders in the context of menopausal complaints. Osteoporosis is a further symptom that can go along with menopause. Studies in rat models also indicate a positive effect of *A. racemosa* extracts on osteoporosis (Wuttke et al. 2014; Guo et al. 2017; Henneicke-von Zepelin 2017; Drewe et al. 2022).

As extracts from rhizomes and roots of *A. racemosa* are established and show significant effects in the treatment of climacteric symptoms, investigations on their active compounds have been made. Chemical characterization of *A. racemosa* revealed the triterpene glycosides with a cycloartane skeleton (e.g., actein) to be the major recognized group. Additionally, further aglyca of triterpenoids have been identified by Qiu et al. (2014), namely e.g., acetol, cimigenol, hydroshengmanol, cimiracemoside, and others. Besides, a wide range of 73 nitrogen-containing metabolites have been isolated, like betains, guanidines, derivatives of choline or pyridoxine, and several alkaloids. Hydroxycinnamic acids and their esters are the third group of important natural products in this genus (Gödecke et al. 2009; Nikolić et al. 2012; Guo et al. 2017).

The benefit of *A. racemosa* extracts has been mostly related to the triterpenoids (Leach and Moore 2012). Investigations on biological activities described these compounds to have anti-proliferative, anti-viral, and anti-tumor effects (Watanabe et al. 2002; Sakurai et al. 2003, 2004). Furthermore, the hydroxycinnamic acid esters (cimicifugic acids and fukinolic acid) were reported to have anti-tumor, anti-viral, anti-oxidative, anti-inflammatory, estrogenic, vasoactive, and collagenolytic activities. The inhibition of cytochrome P450 enzymes and hyaluronidase, influence on plant development, and suppression of allergic reactions have also been attributed to fukinolic acid and/or cimicifugic acids (Jahn and Petersen (2022) and literature cited herein).

**Fig. 1:**
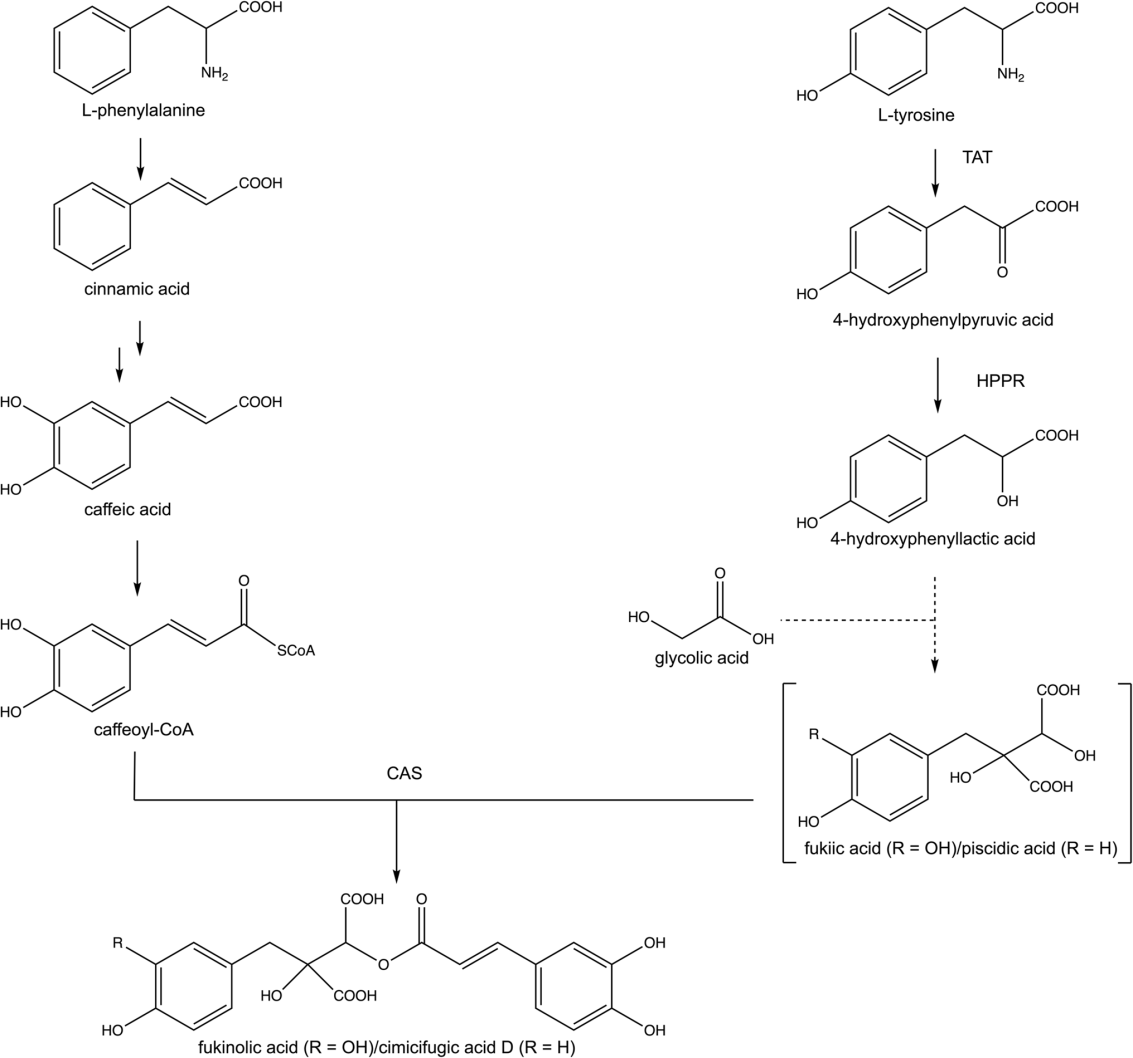
Hypothetical pathway of fukinolic acid biosynthesis. The formation of fukinolic acid arises from l-tyrosine and l-phenylalanine (Hasa and Tazaki 2004). l-phenylalanine is converted into caffeoyl-CoA, representing the caffeic acid moiety of fukinolic acid. l-Tyrosine is converted to 4-hydroxyphenylpyruvic acid by tyrosine aminotransferase (TAT) and further reduced to 4-hydroxyphenyllactic acid by hydroxyphenylpyruvic acid reductase (HPPR). Addition of glycolic acid, possibly activated as glycolyl-CoA, results in the benzyltartaric acid part (piscidic and/or fukiic acid). Transesterification with hydroxycinnamoyl-CoA by cimicifugic acid synthase (CAS) (Werner and Petersen 2019) results in fukinolic acid and/or cimicifugic acids and coenzyme A

The biosynthesis of cimicifugic and fukinolic acids has been investigated in *Petasites japonicus* (Asteraceae) by ^13^C-labeling experiments suggesting that l-tyrosine and l-phenylalanine are the precursors of fukinolic acid (Hasa and Tazaki 2004). Fukinolic acid is composed of two parts: a caffeic acid moiety and a benzyltartaric acid moiety. The caffeic acid moiety originates from caffeoyl-CoA that emerged from l-phenylalanine. The benzyltartaric acid moiety arises from l-tyrosine, that is converted into 4-hydroxyphenylpyruvic acid at first and afterwards into fukiic acid (the benzyltartaric acid moiety). The esterification of fukiic acid and caffeoyl-CoA (derived from l-phenylalanine) finally results in fukinolic acid. However, according to the postulated fukiic acid biosynthetic pathway based on the incorporation of l-tyrosine and acetic acid, the source of one OH-group, that is necessary for esterification, remains unclear. As the well-established rosmarinic acid (RA) biosynthesis pathway (Petersen et al. 1993) and the proposed fukiic acid pathway seem to share a few metabolites, we assume, that hydroxyphenylpyruvic acid reductase (HPPR) may be involved in fukinolic acid biosynthesis (Fig. 1).

Hydroxyphenylpyruvic acid reductase (HPPR) has mostly been described as an enzyme of RA biosynthesis (e.g., from *Coleus blumei*) although it was also detected and characterized from *Arabidopsis thaliana* which does not accumulate measurable amounts of RA (Häusler et al. 1991; Xu et al. 2018). β-hydroxypyruvic acid reductase (HPR), on the other hand, is related to photorespiration and is present in several isoforms, e.g., a peroxisomal and a cytosolic isoform (Timm et al. 2008, 2011). It has been proposed that the cytosolic isoform of HPR and the cytosolically localized HPPR may be the same or closely related enzymes since the HPPR enzymes characterized in relation to RA biosynthesis accept both, NADH and NADPH, as cosubstrates and reduce pHPP as well as β-hydroxypyruvic acid (Janiak 2007; Janiak et al. 2010; Busch 2022). On the other hand, HPR1 reported to be peroxisomal (although missing the typical targeting motif SKL and not predicted to be localized in peroxisomes) from *A. thaliana* only accepts β-hydroxypyruvic acid and prefers NADH (Xu et al. 2018).

First investigations to describe biosynthetic steps toward cimicifugic acids have been recently successful (Werner and Petersen 2019). A hydroxycinnamoyl-CoA:piscidic acid hydroxycinnamoyltransferase (syn. cimicifugic acid synthase) has been isolated from *A. racemosa*. This enzyme catalyzes the transfer of a hydroxycinnamoyl moiety from hydroxycinnamoyl-CoA to piscidic acid. By this reaction, cimicifugic acids D, E, J, and K have been generated from piscidic acid in combination with the respective hydroxycinnamoyl-CoA (caffeoyl-, feruloyl-, sinapoyl-, 4-coumaroyl-CoA). Nevertheless, further molecular and biochemical investigations are needed in the context of fukinolic acid and cimicifugic acid biosynthesis. We here describe the isolation and characterization of a hydroxy(phenyl)pyruvic acid reductase (H(P)PR) from *A. racemosa* which can qualify to be involved in the formation of the benzyltartaric acid moiety.

## Materials and methods

### Plant material

A callus culture from *A. racemosa* has been initiated from leaves and shoots of *A. racemosa* plants purchased in 2015 from Rühlemann’s Kräuter & Duftpflanzen, Horstedt, Germany (CIM01) and grown in the Old Botanical Garden, Philipps-Universität Marburg. The plant material was pre-sterilized with ethanol for approximately 1 min followed by sterilization in 10% commercial bleach for 5–15 min. After washing in sterile water for three times, the plant material was cut and placed on MS medium (Murashige and Skoog 1962) containing 3% sucrose, 1 µM naphthaleneacetic acid, and 0.5 µM thidiazuron solidified with 0.8% agar (Lata et al. 2002) (MSTDZ medium). Growing undifferentiated callus cells were further cultivated on MSTDZ medium under continuous light (~ 15 µmol photons m^−2^ s^−1^) at 23 ± 2 °C and transferred regularly every 6 weeks. Suspension cultures were established by transferring 5 g callus material into a 100 ml Erlenmeyer flask containing 50 ml liquid MSTDZ medium. The flasks were incubated at 23 ± 2 °C under shaking (100 rpm) and continuous light (~ 15 µmol photons m^−2^ s^−1^). For maintenance of cultures, 5 g suspension-cultured cells were transferred weekly into fresh 50 ml MSTDZ medium.

### Isolation of RNA and cDNA synthesis

Cells from a 4-day-old suspension culture were separated from the medium by vacuum filtration. 15–20 mg cell material pulverized in liquid nitrogen were used for mRNA extraction as described by Chomczynski and Sacchi (1987). Integrity of RNA was checked by agarose gel electrophoresis and the concentration (A_260_) and purity (A_260_/A_280_) assessed photometrically. cDNA was synthesized from mRNA with the RevertAid First-Strand cDNA Synthesis Kit (ThermoScientific).

### Amplification of ArH(P)PR and homologous recombination

We searched the 1kP database (https://db.cngb.org/onekp/) for HPPR in *A. racemosa* on the basis of amino acid sequence identity toward the fully characterized HPPR from *C. blumei* (CAD47810; 82% identity). The retrieved coding sequence CYVA-2075208 was chemically synthesized by Eurofins Genomics, ligated into pDrive and transferred into *E. coli* EZ. After sequence verification (Microsynth Seqlab), the plasmid was used for PCR to add overhangs complementary to pET15b for introduction into the plasmid by homologous recombination in *E. coli* SoluBL21 (Jacobus and Gross 2015). Additionally, the putative ArH(P)PR sequence was amplified with full-length primers using cDNA as template (Genbank OR393286) for verification of expression. For primers and amplification conditions, see Suppl. Tables S1 and S2; primers were obtained from Eurofins Genomics.

Products resulting from PCR or restriction digest were analyzed by agarose gel electrophoresis (1.0%) in 1xTAE-buffer (40 mM Tris, 20 mM acetic acid, 1 mM EDTA). Bands with the expected length were extracted (NucleoSpin Gel and PCR clean-up kit, Machery-Nagel) for further processing.

### Gene expression analysis

Verification of expression of ArH(P)PR was performed by PCR using cDNA isolated from various plant parts (roots, leaves, stems, flower stalks, flowers). A partial sequence (for primers see Suppl. Table S1) was amplified using different amounts of cDNA. PCR conditions are listed in Suppl. Table S2. PCR products were visualized by agarose gel electrophoresis.

### Expression of ArH(P)PR and purification

*E. coli* SoluBL21 containing pET15b::ArH(P)PR was cultivated in overnight cultures in LB media with ampicillin (100 µg/ml) (37 °C, 220 rpm) which were then inoculated into TB media with ampicillin (100 µg/ml) (for media, see Lessard 2013). The cultures were incubated at 37 °C and 220 rpm until the optical density at 600 nm (OD_600_) was appr. 0.4. After induction with 1 mM isopropyl-β-d-1-thiogalactopyranoside, bacteria were cultivated overnight (25 °C, 220 rpm). Bacteria were harvested by centrifugation (4 °C, 6000 g, 10 min) and the cells frozen at -80 °C. Thawed cells were resuspended in 0.1 M potassium phosphate buffer (KPi = 0.1 M KH_2_PO_4_/K_2_HPO_4_) pH 7.5 (4 ml KPi buffer/g pellet) and incubated with appr. 50 mg lysozyme for 30 min on ice, followed by ultrasonication (4 °C, 4 × 15 s). The suspension was centrifuged for the collection of the supernatant (4 °C, 6000 g, 10 min). The crude extract was used for purification of 6xHis-tag-containing ArH(P)PR by Ni–NTA chromatography (Novagen). The column was equilibrated with binding buffer (50 mM KPi buffer pH 8.0, 10 mM imidazole, 300 mM NaCl). The crude extract was adjusted to 10 mM imidazole, 300 mM NaCl, applied to the column containing 1 ml Ni–NTA agarose material, incubated for 1 h on ice and the flow-through collected for SDS-PAGE. Washing was done with 12 ml wash buffer (50 mM KPi pH 8.0, 20 mM imidazole, 300 mM NaCl) before elution of the protein with 3 ml elution buffer (50 mM KPi pH 8.0, 50 mM imidazole, 300 mM NaCl). Washing and elution fractions were collected for SDS-PAGE. The eluted His-tagged protein was then loaded onto a PD10-column (GE Healthcare) for desalting and transfer into 0.1 M KPi buffer pH 7.5. The protein concentration of purified ArH(P)PR was determined (Bradford 1976) and aliquots were stored at -80 °C.

### SDS-PAGE and Western-blot analysis

Proteins from fractions gathered from Ni–NTA chromatography were separated by SDS-PAGE (Laemmli 1970) followed by Western-blot analysis of 6xHis-tagged proteins. The gel was either dyed with Coomassie Brilliant Blue R250 or transferred onto a PVDF membrane using the Towbin et al. (1979) buffer system. Immunodetection was carried out with mouse anti-6xHis-tag monoclonal antibodies (ThermoFisher; MA1-21,315) as primary and goat anti-mouse antibody conjugated with alkaline phosphatase (Life Technologies; A16087) as secondary antibody. A solution with nitro blue tetrazolium chloride and 5-brom-4-chloro-3-indolyl-phosphate was used for visualization (https://www.sysy.com/protocols/westernblot-ap-detection). Proteins conjugated with a 6xHis-tag show up with a purple-colored band.

### Photometric assays for ArH(P)PR

Enzymatic activity was measured in a pre-tempered double-beam photometer (Analytik Jena) measuring the oxidation of NAD(P)H. As the substrate 4-hydroxyphenylpyruvic acid (pHPP) showed an absorption at 340 nm, the wavelength for all activity measurements was shifted to 380 nm. Extinction coefficients for NADH (ε = 960.67 l cm^−1^ mol^−1^) and NADPH (ε = 1114.83 l cm^−1^ mol^−1^) at 380 nm were determined in 0.1 M KPi buffer pH 7.5. The pH-optimum was determined in Britton–Robinson buffers (pH 4.0–10.0) and the temperature optimum in 0.1 M KPi buffer pH 7.5. For the determination of all parameters, three biological replicates were measured two–three times each.

For Michaelis–Menten kinetics, the following (co-)substrates were investigated: NADPH, NADH, 4-hydroxyphenylpyruvic acid (pHPP), 3,4-dihydroxyphenylpyruvic acid (DHPP), phenylpyruvic acid (PP), pyruvic acid (P), and β-hydroxypyruvic acid (β-HP). The enzyme assays for substrate kinetics were composed of 1 mM NADPH, 20 µl ArH(P)PR (0.328–0.412 mg/ml), and 0.1 M KPi buffer pH 7.5 with pHPP, DHPP, PP, P, or β-HP in 1 ml reaction volume. Final concentrations of substrates and cosubstrates are given in Suppl. Table S3. Depending on the substrate, the reaction time was 3–10 min. Kinetic data were analyzed with the GraphPad Prism 8 software using the Michaelis–Menten model.

### Sample preparation for LC–MS

Enzyme assays for LC–MS analysis were prepared as above but incubated for 30 min and stopped with 50 µl 6 M HCl. For analysis of aromatic products, the samples were extracted with 500 µl EtOAc twice, the organic solvent evaporated and the residues re-dissolved in 100 µl 50% MeOH. For the detection of polar substrates, the aqueous enzyme assay was evaporated and re-dissolved in 200 µl 50% MeOH. LC–MS analysis was performed in the negative mode with an Agilent 1260 system with diode array detector (190–400 nm) coupled with a micrOTOF-Q III with ESI source. For elution 0.1% aqueous formic acid (A) and 0.1% formic acid in acetonitrile (B) were mixed as follows: 0–10 min 5% B to 100% B, 10–15 min 100% B, 15–20 min 100% B to 5% B. Measurement was done at 25 °C on a Multospher 120 RP18 column (250 × 2 mm, 5 μm, CS-Chromatographie Service) with a flow rate of 0.5 ml/min. For the detection of β-hydroxypyruvic acid and pyruvic acid, the samples were measured by MS only.

### Phylogenetic analysis

As there is—to our best knowledge—no available sequence of HPPR in the family Ranunculaceae, the translated amino acid sequence of ArH(P)PR was aligned with further amino acid sequences from species of various plant families. 62 predicted HPPR sequences were collected, and a Maximum Likelihood tree (1000 bootstraps) created with the MEGA 11 software (Tamura et al. 2021). The amino acid sequences were taken from UniProt, NCBI, arabidopsis.org, Phytozome13, The Rice Annotation Project Database and BLAST. Additionally, we included 23 putative HPR sequences for the differentiation between HPR and HPPR.

## Results

### Isolation of a cDNA sequence encoding ArH(P)PR

Searching the 1kP database (https://db.cngb.org/onekp/) of *A. racemosa* with the well-characterized *Coleus blumei* HPPR (CAD47810) revealed a potential H(P)PR sequence. The identity of these two amino acid sequences was at 82%. Consequently, the corresponding nucleotide sequence was chemically synthesized for protein formation in *E. coli*. Additionally, full-length PCR primers directed against this HPPR-like sequence were designed. After the successful isolation of RNA from *A. racemosa*, the full-length coding sequence (945 bp) was amplified by PCR. This sequence encodes a protein with 314 amino acid residues with a calculated mass of 34.34 kDa.

The alignment with the fully characterized H(P)PR from *C. blumei* showed an identity of 82%. Amino acid residues identified to be essential for substrate binding and catalysis are conserved (Suppl. Fig. S1). Searching the same database with this identified putative ArH(P)PR sequence only retrieved other scaffolds with less than 50% identity to the bait sequence.

### Phylogenetic analysis

Amino acid sequences of HPPR and HPR from different plant families were gathered from databases and submitted to a phylogenetic analysis (maximum likelihood, 1000 bootstraps) together with the translated amino acid sequence of ArH(P)PR (Suppl. Fig. S2). To distinguish whether the sequence of ArH(P)PR is closer to HPR or HPPR analogs, 62 putative HPPR sequences in comparison to 23 putative HPR sequences (most of them carrying the peroxisomal targeting sequence SKL) were included. It must be noted that the substrate acceptance of the encoded putative HPPR and HPR enzymes has been tested only in few cases which are indicated in the tree by an asterisk.

The amino acid sequence of ArH(P)PR (Ranunuculaceae) shows highest similarities with HPPR sequences from *Cinnamomum micranthum* (Lauraceae) and *Artemisia annua* (Asteraceae). However, except for the family Lamiaceae where all included HPPR sequences cluster together, a close correlation between sequences from the same family does not show up, e.g., sequences from Asteraceae species also turn up in other branches. Except for a sequence from *Physcomitrium patens* (not carrying the SKL peroxisomal targeting motif) HPRs are grouped in a separated branch, clearly distinct from proven or suggested HPPRs. Overall, the tree suggests that HPPRs within one plant family might have evolved from different ancestors.

### Expression and biochemical characterization of ArH(P)PR

The full-length nucleotide sequence of ArH(P)PR was on the one hand chemically synthesized and on the other hand amplified from RNA/cDNA isolated from an *A. racemosa* suspension culture (Suppl. Fig. S3) and an *N*-terminal 6xHis-tag was attached by introduction into the expression vector pET15b by homologous recombination. *E. coli* SoluBL21 was transformed and cultivated for protein production. ArH(P)PR was purified by metal chelate chromatography and samples were analyzed by SDS-PAGE and Western blot. As a control, bacteria harboring the empty vector pET15b were treated in the same way. After incubation with anti-6xHis-tag antibodies, Western blots showed a protein around 40 kDa in bacteria containing pET15b::ArH(P)PR (ArH(P)PR = 36.51 kDa including 6xHis-tag), which is absent in the empty vector control (Suppl. Fig. S4).

Catalytic activities of ArH(P)PR were determined photometrically with purified protein by recording the oxidation of the cosubstrate NAD(P)H. Proteins from empty vector (pET15b) controls were not active (Suppl. Fig. S5). The pH-optimum of ArH(P)PR was determined to be between pH 7.32 and 7.77 (Suppl. Fig. S6a). Thus, a 0.1 M KPi-buffer with pH 7.5 was chosen for kinetic analyses. The optimal reaction temperature was at 38 °C (Suppl. Fig. S6b).

**Fig. 2:**
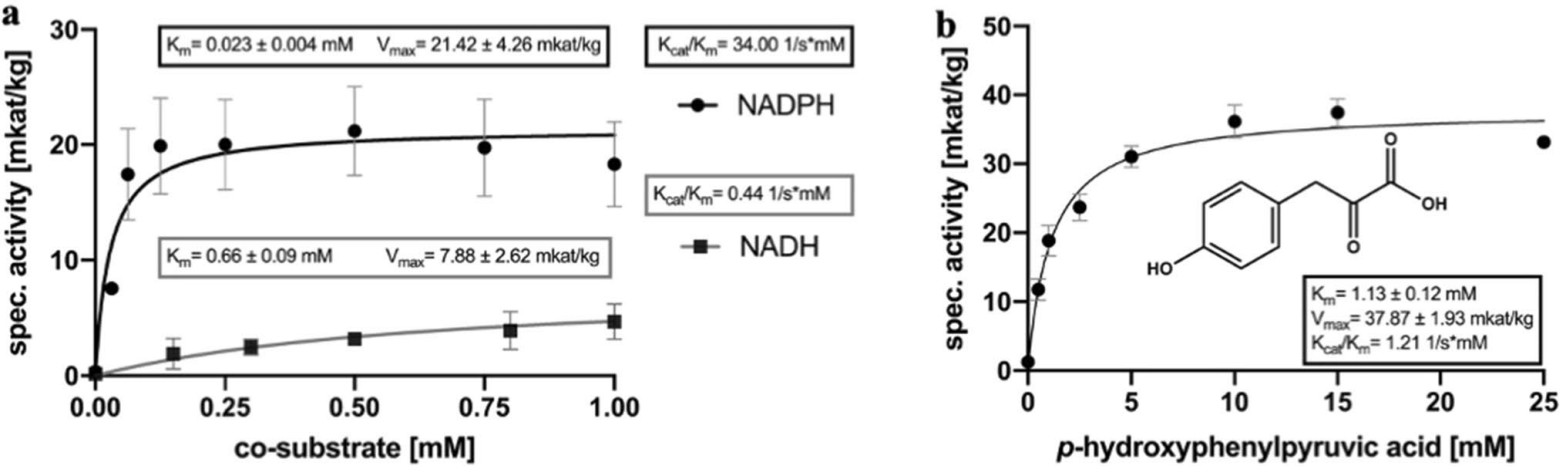
Substrate saturation curves for NADPH and NADH (**a**) and 4-hydroxyphenylpyruvic acid (**b**). **a** Michaelis–Menten graphs for NADH and NADPH at final concentrations of 0–1.5 mM (data only shown up to 1 mM due to high background absorption of the cosubstrates); mean values ± SD (NADH, *n* = 3 × 3; NADPH, *n* = 4 × 3). **b** Michaelis–Menten graph for 4-hydroxyphenylpyruvic acid (0–25 mM) together with 1 mM NADPH; mean values ± SD (*n* = 4 × 3)

The Michaelis–Menten analysis of both cosubstrates showed a considerably higher affinity of ArH(P)PR for NADPH compared to NADH (Fig. 2a and Table 1). The K_m_ was at 0.023 ± 0.004 mM for NADPH and at 0.66 ± 0.09 mM for NADH. Additionally, the catalytic efficiency with NADPH (K_cat_/K_m_ 34.00 1/s mM) was about 80-fold higher than with NADH (K_cat_/K_m_ 0.44 1/s mM). Consequently, NADPH was used as cosubstrate for further kinetic analyses.

**Table 1:** Kinetic data for ArH(P)PR determined by Michaelis–Menten graphs**,** mean value ± SD, except the values for β-HP (mean value ± SEM, *n* = 4 × 3)

Substrate	K_m_ [mM]	V_max_ [mkat/kg]	K_cat_ [1/s]	K_cat_/K_m_ [1/s mM]
pHPP	1.13 ± 0.12	37.87 ± 1.93	1.36 ± 0.07	1.21
β-HP	0.26 ± 0.12	18.47 ± 4.31	0.67 ± 0.16	2.59
DHPP	23.99 ± 4.65	402.50 ± 72.51	14.70 ± 2.69	0.61
P	1.89 ± 0.51	11.72 ± 1.96	0.43 ± 0.06	0.23
PP	5.32 ± 2.77	11.53 ± 1.96	0.43 ± 0.07	0.08
NADPH	0.023 ± 0.004	21.42 ± 4.26	0.78 ± 0.16	34.00
NADH	0.66 ± 0.09	7.88 ± 2.62	0.29 ± 0.09	0.44

pHPP, 4-hydroxyphenylpyruvic acid, n = 4 × 3; *β*-HP, *β*-hydroxypyruvic acid, n = 4 × 3; DHPP, 3,4-dihydroxyphenylpyruvic acid, n = 4 × 3; P, pyruvic acid, n = 4 × 3; PP, (phenylpyruvic acid, n = 4 × 3; NADP, n = 4 × 3; NADH, n = 3 × 3

HPPR converts 4-hydroxyphenylpyruvic acid (pHPP) to 4-hydroxyphenyllactic acid (pHPL). Substrate saturation curves were recorded with saturating 1 mM NADPH and 0–25 mM pHPP. The calculated K_m_ value for pHPP was at 1.13 ± 0.12 mM (Fig. 2b and Table 1), the V_max_ value was at 37.87 ± 1.93 mkat/kg and the catalytic efficiency (K_cat_/K_m_) at 1.21 1/s mM.

Additional substrates were tested for their acceptance: 3,4-dihydroxyphenylpyruvic acid (DHPP), β-hydroxyphenylpyruvic acid (β-HP), phenylpyruvic acid (PP), pyruvic acid (P) and 4-hydroxy-3-methoxyphenylpyruvic acid (4H3MPP). All mentioned compounds could serve as substrate for ArH(P)PR. Apart of 4H3MPPA (due to a reduced availability of the substrate), substrate saturation curves were recorded for all substrates.

**Fig. 3:**
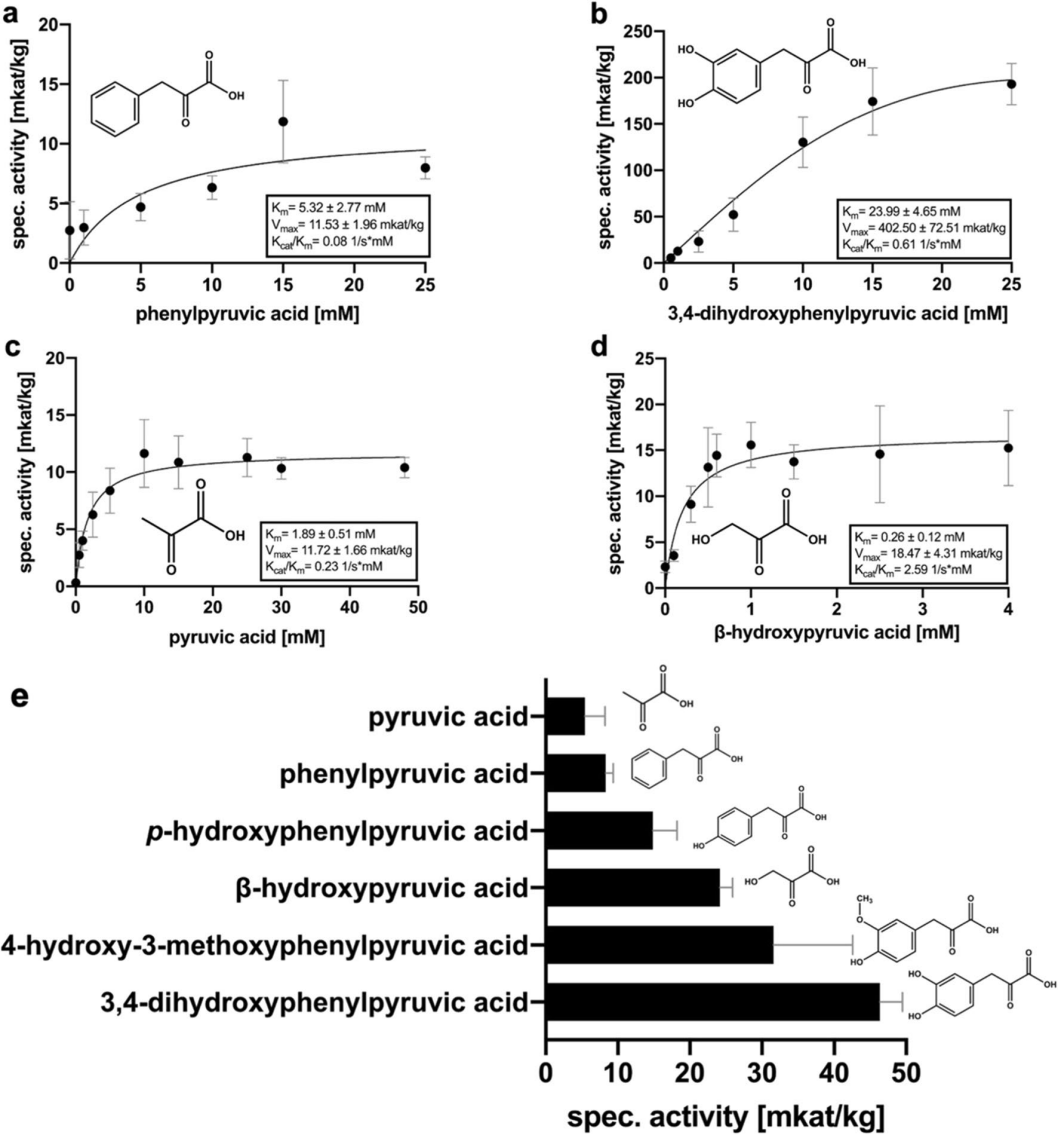
Substrate saturation curves (**a–d**) and comparison of specific activities of ArH(P)PR for different substrates (**e**). **a** Phenylpyruvic acid (*n* = 4 × 3, mean value ± SD); **b** 3,4-dihydroxyphenylpyruvic acid (*n* = 4 × 3, mean value ± SD); **c** pyruvic acid (*n* = 4 × 3, mean value ± SD); **d** β-hydroxypyruvic acid (*n* = 4 × 3, mean value ± SE). **e** Comparison of specific activities of ArH(P)PR using 0.5 mM as final concentration of each substrate (*n* = 3 × 3, mean value ± SD)

β-HP was the substrate accepted with the highest affinity (K_m_ 0.26 ± 0.12 mM) and the highest catalytic efficiency (K_cat_/K_m_ of 2.59 1/s mM) with a V_max_ of 18.47 ± 4.31 mkat/kg and showed a very strong substrate inhibition at β-HP concentrations exceeding 1 mM (Suppl. Fig. S7). After pHPP as second-best substrate (see above), P was accepted with a K_m_ of 1.89 ± 0.51 mM followed by PP (K_m_ 5.32 ± 2.77 mM) and DHPP (K_m_ 23.99 ± 4.65 mM). Due to the exceptionally high V_max_ with DHPP (V_max_ 402.50 ± 72.51 mkat/kg; K_cat_/K_m_ 0.61 1/s mM), the catalytic efficiency with this substrate was higher than for P (V_max_ 11.72 ± 1.66 mkat/kg, K_cat_/K_m_ 0.23 1/s mM) and PP (V_max_ 11.53 ± 1.96 mkat/kg, K_cat_/K_m_ 0.08 1/s mM) (Fig. 3 and Table 1).

### Verification of products by LC–MS

**Fig. 4:**
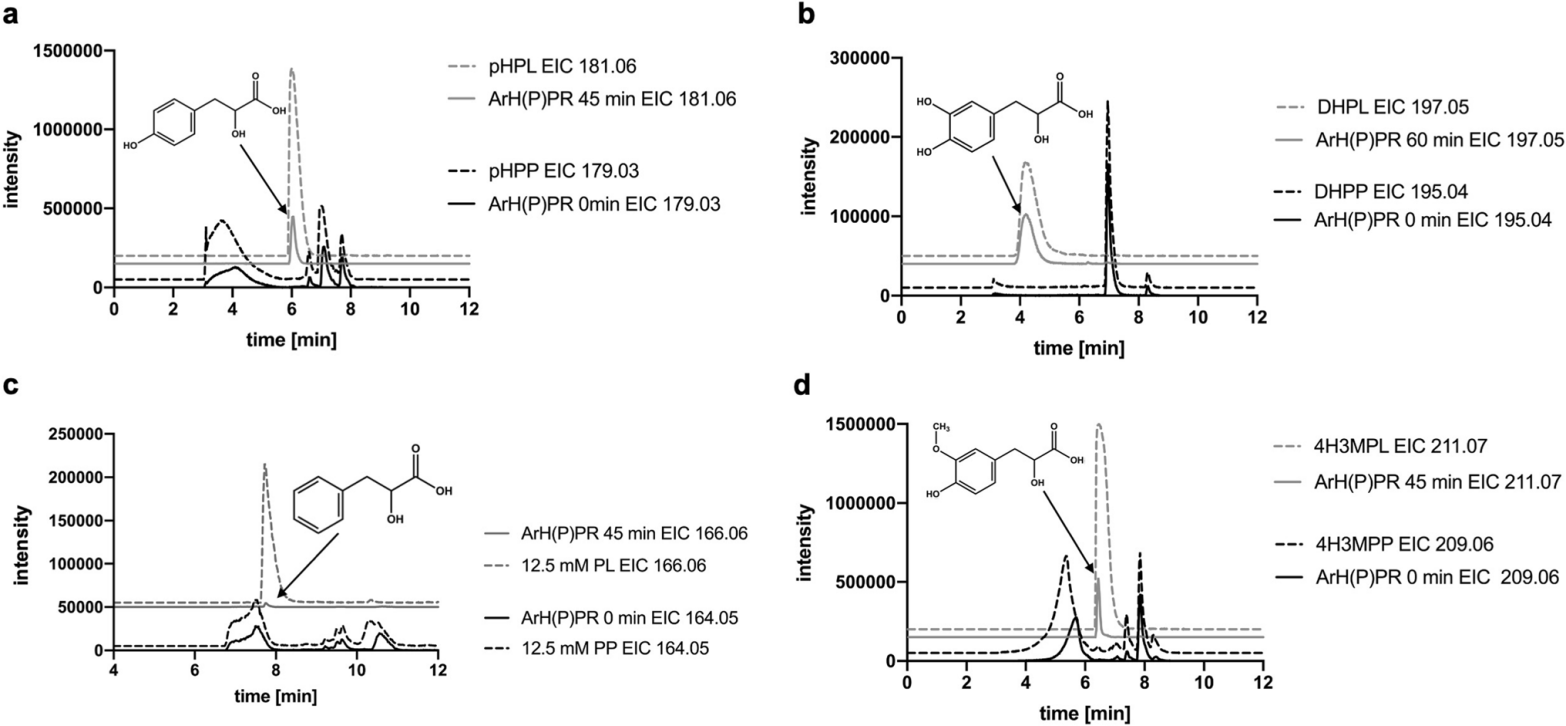
Verification of product formation of ArH(P)PR with NADPH and different substrates by LC–MS. Black line = control, black dashed line = 12.5 mM standard of educt, grey line = incubated enzyme assay, grey dashed line = 12.5 mM standard for product. **a** ArH(P)PR with phenylpyruvic acid (PP). **b** ArH(P)PR with 4-hydroxyphenylpyruvic acid (pHPP). **c** ArH(P)PR with 4-hydroxy-3-methoxyphenylpyruvic acid (4H3MPPA). **d** ArH(P)PR with 3,4-dihydroxyphenylpyruvic acid (DHPP)

The enzymatically formed products were analyzed by LC–MS for verification. For pHPP (*m/z* 179.03 [M–H]^−^) as substrate, the detection of 4-hydroxyphenyllactic acid (pHPL; *m/z* 181.06 [M–H]^−^) was successful (Fig. 4a). The catalytic activity of ArH(P)PR with DHPP (*m/z* 195.04 [M–H]^−^) was verified by the formation of 3,4-dihydroxyphenyllactic acid (DHPL; *m/z* 197.05 [M–H]^−^) (Fig. 4b). The presence of *m/z* 166.06 [M–H]^−^ (PL; phenyllactic acid) confirmed the reduction of PP (Fig. 4c). The conversion of 4H3MPP (*m/z* 209.06 [M–H]^−^) as substrate lead to 4-hydroxy-3-methoxyphenyllactic acid (4H3MPL) as product with a mass of *m/z* 211.07 [M–H]^−^ (Fig. 4d).

Due to the used instruments, detection of substrates and products with low molecular masses proved to be problematic. The detection of P and its putative enzymatically formed product lactic acid was not possible. Tests with β-HP (*m/z* 103.02 [M–H]^−^) as substrate were only analyzed mass-spectrometrically. The substrate could be detected in the control at *m/z* 103.02 [M–H]^−^ (Suppl. Fig. S8). The 30-min incubated assay showed a mass of *m/z* 105.02 [M–H]^−^ indicating glyceric acid formation.

### Expression of ArH(P)PR in plant material

To show in which plant parts the ArH(P)PR gene is expressed, cDNA from RNA extracted from roots, leaves, stems, flower stalks, and flowers was used to amplify partial sequences from ArH(P)PR. Expression could be demonstrated in above-ground plant parts (leaves, stems, flower stalks, and flowers; data not shown).

## Discussion

After having gained insight into one step of cimicifugic acid biosynthesis in *A. racemosa* recently, namely the transesterification of a hydroxycinnamoyl-CoA with piscidic acid by cimicifugic acid synthase (Werner and Petersen 2019), we are now interested in the biosynthesis of piscidic and fukiic acids (the benzyltartaric acid moieties). For the biosynthesis of fukinolic acid, a mechanism has been postulated by Hasa and Tazaki (2004). This mechanism proposed the benzyltartaric acid moiety of fukinolic acid to arise from l-tyrosine and acetic acid. The postulated pathway toward fukiic and piscidic acids shares some reactions with the formation of the 3,4-dihydroxyphenyllactic acid part of RA, namely the formation of 4-hydroxyphenylpyruvic acid by transamination of l-tyrosine and the following reduction to 4-hydroxyphenyllactic acid (Petersen et al. 1993). In the feeding experiments of Hasa and Tazaki (2004), [1,2-^13^C_2_] sodium acetic acid was incorporated into fukiic acid at positions 1 and 2 and [1-^13^C] l-tyrosine with the label in position 5 (Fig. 1). Thus, the authors proposed a condensation of a phenylpropane with acetyl-CoA. This, however, does not explain how the second aromatic hydroxyl group and—more importantly—the hydroxyl group in position 2 which is necessary for esterification with hydroxycinnamoyl-CoA arise. Taking this into account, glycolyl-CoA could be the precursor added to a phenyllactic acid derivative which would result in the necessary OH-group. Glycolyl-CoA was shown to be formed biochemically by (hydroxy)pyruvic acid dehydrogenase converting β-hydroxypyruvic acid instead of pyruvic acid (Vamecq and Poupaert 1990). The same authors also showed that glycolyl-CoA can be added to oxaloacetic acid by citric acid synthase. This could be a reaction scheme for the addition of the glycolyl moiety from glycolyl-CoA to phenyllactic acid. For the formation of the hydroxycinnamoyl moiety of fukinolic and cimicifugic acids, l-phenylalanine is converted to 4-coumaroyl-CoA by the reactions of the phenylpropanoid pathway, eventually leading to the caffeic acid part of fukinolic acid.

To gain insight into the formation of the possible piscidic/fukiic acid precursor 4-hydroxyphenyllactic acid, we investigated the enzyme reducing pHPP to pHPL in *A. racemosa*. To the best of our knowledge, this is the first report on a H(P)PR from a species of the Ranunculaceae. HPPR (EC 1.1.1.237) has first been described and characterized from *C. blumei* (syn. *Solenostemon scutellarioides*, *Plectranthus scutellarioides*,* C. scutellarioides*) as an enzyme of RA biosynthesis (Petersen and Alfermann 1988; Häusler et al. 1991). Later, the encoding gene was identified and the heterologously expressed enzyme characterized and crystallized for structural analyses (Kim et al. 2004; Janiak et al. 2010). The participation of HPPR in RA biosynthesis has been shown by RNA_i_ inhibition and overexpression experiments (Hücherig and Petersen 2012). The enzyme has additionally been investigated in *Salvia miltiorrhiza*, *A. thaliana*, *Anthoceros agrestis* and *Prunella vulgaris* (Wang et al. 2017; Xu et al. 2018; Busch 2022; Ru et al. 2023). While the function of HPPR is commonly coupled to the formation of RA, Xu et al. (2018) reported that *A. thaliana* does not contain measurable amounts of RA, but nevertheless has HPPRs converting pHPP to pHPL.

The sequence of ArH(P)PR was identified in the 1kP database on the basis of an alignment with the HPPR sequence from *C. blumei* (CAD47810; 82% identity on amino acid level). Other *A. racemosa* sequences returned in this search displayed less than 50% identity. The phylogenetic analysis showed that ArH(P)R grouped together with HPPR from species of the Papaveraceae and Asteraceae. While Papaveraceae and Ranunculaceae both belong to the order Ranunculales, Asteraceae, order Asterales, are phylogenetically quite distant. Hydroxypyruvic acid reductase (HPR) sequences appeared together in a separate branch and, thus, cannot be considered closely related to the HPPR sequences.

It has been discussed recently whether the putatively cytosolically localized HPPR is a genuine enzyme of RA biosynthesis or whether the cytosolic HPR is the same enzyme as HPPR with two activities, reduction of β-hydroxypyruvic to glyceric acid in photorespiration and reduction of pHPP to pHPL in RA biosynthesis and other specialized metabolic pathways (Hücherig and Petersen 2012; Petersen 2013). Since RNA_i_ suppression and HPPR overexpression both showed an impact on RA accumulation in *C. blumei,* the participation of HPPR in RA synthesis is proven (Hücherig and Petersen 2012).

In *A. thaliana*, the peroxisomal HPR1 (At1g68010) is essential for photorespiration and catalyzes the reduction of β-hydroxypyruvic to glyceric acid with NADH as cosubstrate (Timm et al. 2008, 2011). This enzyme was not active with pHPP or NADPH (Xu et al. 2018). Deletion of HPR1 was not lethal in *A. thaliana* which led to the detection of a cytosolic HPR2 (At1g79870) which can overtake the role of HPR1 in photorespiration preferring NADPH as cosubstrate. Even a double mutant of HPR1 and 2 could survive due to a third HPR (At1g12550) which was supposed to be plastidic (Timm et al. 2008, 2011). A triple mutant encompassing the knockout of all three H(P)PR isoforms in *A. thaliana* resulted in a strongly growth-retarded phenotype with reduced photochemical efficiency (Timm et al. 2011). Since HPPR from *C. blumei* (87.2%) and *A. racemosa* (88.9%) share high sequence similarities to HPPR2 from *A. thaliana*, an involvement of both encoded enzymes in photorespiration can be assumed besides the involvement in hydroxyphenyllactic acid formation. Xu et al. (2018) published three putative HPPR sequences from *A. thaliana*. HPPR2 (At1g79870) and HPPR3 (At1g12550) both converted β-HP and pHPP with NADPH as cosubstrate, although HPPR3 showed a pronounced preference for pHPP. HPPR4 (At1g45630) was rather inactive. All three HPPR proteins were localized in the cytoplasm.

Unfortunately, it is not easy to verify the involvement of H(P)PR in piscidic acid formation as well as in photorespiration. *A. racemosa* seeds show very low germination rates (own unpublished experiments) and transformation protocols are not known. In addition, the production of cimicifugic/fukinolic acids in in vitro cultures as well as in natural plants is rather low. Gene expression analyses have, however, shown that ArH(P)PR is expressed in above-ground and, thus, photosynthesizing plant parts which is expected for an enzyme of photorespiration. This result corresponds to the fact that the highest content of fukinolic/cimicifugic acids had been detected in leaves and flowers of *A. racemosa* (Werner and Petersen 2019), which are the same plants as analyzed here.

ArH(P)PR has HPR as well as HPPR activities and showed a significantly higher affinity towards β-HP (K_m_ 0.26 ± 0.12 mM) compared to pHPP (K_m_ 1.13 ± 0.12 mM). Interestingly, β-HP was the only substrate leading to substrate inhibition. With regards to the cosubstrate, ArH(P)PR clearly favors NADPH (K_m_ 0.023 ± 0.004 mM) instead of NADH (K_m_ 0.66 ± 0.09 mM). These results correlate with the results from Janiak (2007), Busch (2022) and Ru et al. (2023). All previously mentioned authors also reported the product formation with pyruvic (P) and phenylpyruvic acids (PP). 4H3MPP and DHPP were additionally described as accepted substrates by Janiak (2007) and Busch (2022). The highest catalytic efficiencies (K_cat_/K_m_) for ArH(P)PR were recorded for β-HP followed by pHPP with about half the level of β-HP. The overall catalytic efficiencies were in the order β-HP > pHPP > DHPP > P > PP. Almost the same order was observed for the substrate affinities: β-HP > pHPP > P > PP > DHPP.

Since the overall identity of ArH(P)PR and HPPR from *C. blumei* with regards to the amino acid sequences is high at 82% with a similarity of 91%, we deduced the reactive site and binding motifs of ArH(P)PR on basis of the crystal structures of HPPR from *C. blumei* (pdb: 3BA1, 3BAZ) (Janiak et al. 2010). The NAD(P)H/NAD(P)^+^ binding motif is well conserved in CbHPPR and ArH(P)PR. As observed in CbHPPR, the Asp residue in position 275 (276 in ArH(P)PR) is replaced by Ser which enables NADPH as well as NADH binding. Other amino acid residues essential for cosubstrate fixation such as Ile230, Arg256 and His279 (Ile231, Arg257, His280 in ArH(P)PR) are conserved as well. The catalytic site in CbHPPR is formed by Val76, Gly77, Arg232, Glu261 and His279. These are conserved in ArH(P)PR as Val76, Gly77, Arg233, Glu262 and His280 (Suppl. Fig. S1).

Our investigations showed that ArH(P)PR can readily be involved in the formation of pHPP in the biosynthetic pathway towards fukiic and piscidic acids. However, a main or additional role in photorespiration seems probable.

## Conclusion

Piscidic acid and fukiic acid are precursors for the biosynthesis of fukinolic and cimicifugic acids in *A. racemosa* (Ranunculaceae). The identification of hydroxyphenylpyruvic acid reductase (H(P)PR) in this species might give a first insight into the possible pathway toward piscidic and fukiic acid. ArH(P)PR cDNA was expressed, and the encoded enzyme biochemically characterized. ArH(P)PR showed a distinct affinity for NADPH as a cosubstrate and preferred β-hydroxypyruvic acid, followed by 4-hydroxyphenylpyruvic, pyruvic, phenylpyruvic and 3,4-dihydroxyphenylpyruvic acids. Thus, ArH(P)PR has HPPR as well as HPR characteristics and might be involved in photorespiration as well as in piscidic/fukiic acid biosynthesis.

## Data Availability

Sequence data were submitted to GenBank under the accession number OR393286.

## Abbreviations

β-HP: β-Hydroxypyruvic acid
H(P)PR: Hydroxy(phenyl)pyruvic acid reductase
DHPL/DHPP: 3,4-Dihydroxyphenyllactic acid/3,4-dihydroxyphenylpyruvic acid
4H3MPL/4H3MPP: 4-Hydroxy-3-methoxyphenyllactic acid/4-hydroxy-3-methoxyphenylpyruvic acid
P: Pyruvic acid
pHPL/pHPP: 4-Hydroxyphenyllactic acid / 4-hydroxyphenylpyruvic acid
PL/PP: Phenyllactic acid/phenylpyruvic acid
RA: Rosmarinic acid
